# Ternary superhydrides for high-temperature superconductivity at low pressures

**DOI:** 10.1093/nsr/nwae003

**Published:** 2024-01-04

**Authors:** Pengfei Shan, Liang Ma, Jinguang Cheng

**Affiliations:** Beijing National Laboratory for Condensed Matter Physics and Institute of Physics, Chinese Academy of Sciences, China; School of Physical Sciences, University of Chinese Academy of Sciences, China; Beijing National Laboratory for Condensed Matter Physics and Institute of Physics, Chinese Academy of Sciences, China; Key Laboratory of Materials Physics, Ministry of Education, School of Physics and Microelectronics, Zhengzhou University, China; Beijing National Laboratory for Condensed Matter Physics and Institute of Physics, Chinese Academy of Sciences, China; School of Physical Sciences, University of Chinese Academy of Sciences, China

## Abstract

Focusing on the ternary hydrides, the new hope of Room-Temperature Superconductivity, this perspective delves into the research background, highlights current challenges, and illuminates promising avenues for future studies.

The quest for room-temperature superconductivity (RTSC) has been a longstanding pursuit in physics since the discovery of superconductivity in 1911. According to the conventional Bardeen-Cooper-Schrieffer theory, atomic metallic hydrogen holds great promise for achieving this goal due to its high Debye temperature and strong electron-phonon coupling. However, metallization of solid hydrogen remains uncertain at pressures up to 400 GPa, the current upper limit of static high-pressure techniques. In 2004, Ashcroft proposed an alternative approach to realize high-Curie-temperature (*T_c_*) superconductivity in hydrogen-rich materials (hydrides) at attainable pressures due to the chemical precompression effect [1]. This concept has stimulated extensive theoretical and experimental investigations into hydride superconductors, for which significant advancements have been achieved in recent years, reigniting the hope of realizing RTSC.

In terms of the chemical bonding around hydrogen, the experimentally discovered high-*T*_c_ hydrides can be divided into two categories [2]: (i) the covalent-type H_3_S, initially reported by the Eremets group in 2015, marking a groundbreaking achievement in the superconducting hydrides with *T*_c_ exceeding 200 K, which for the first time surpasses the *T*_c_ record of cuprate superconductors; (ii) the ionic-type clathrate superhydrides represented by LaH_10_ and CaH_6_. Among them, LaH_10_ currently holds the highest reproducible *T*_c_ of 250–260 K reported thus far. The successful syntheses and characterizations of many binary hydrides under megabar pressures have been made feasible only recently due to the utilization of solid hydrogen sources like BH_3_NH_3_. This advancement has paved the way for extensive explorations of binary superhydrides of most elements in the periodic table, resulting in the discoveries of the following remarkable binary hydride superconductors with *T*_c_ exceeding 100 K [3], i.e. LaH_10_ (*T*_c_ = 250 K @ 170 GPa), YH_9_ (*T*_c_ = 243 K @ 201 GPa), YH_6_ (*T*_c_ = 220 K @ 183 GPa), CaH_6_ (*T*_c_ = 215 K @ 172 GPa), ThH_10_ (*T*_c_ = 159 K @ 174 GPa), ThH_9_ (*T*_c_ = 146 K @ 170 GPa), CeH_10_ (*T*_c_ = 115 K @ 95 GPa), and CeH_9_ (*T*_c_ = 100 K @ 130 GPa), as shown in Fig. 1.

**Figure 1.**
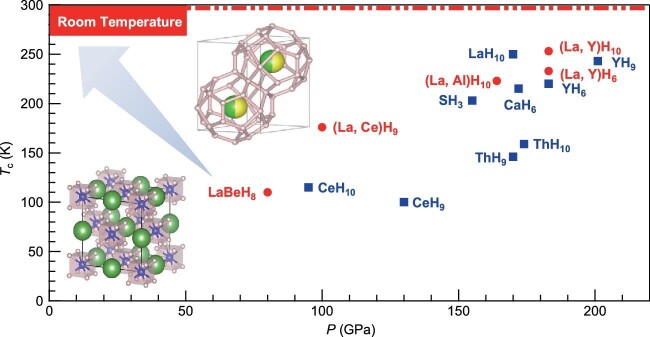
Binary (bule square) and ternary (red circle) hydride superconductors with *T*_c_ >100 K synthesized in experiments; the inset shows the crystal structures of (La, Ce)H_9_ and LaBeH_8_, respectively. The arrow represents the ultimate goal of ‘Towards the RTSC at ambient pressure’.

Although the pressures stabilizing these high-*T*_c_ binary superhydrides ( >∼150 GPa) are considerably lower than that is needed to metallize pure hydrogen, the over–megabar-pressure experiments remain quite challenging. As such, much effort has now been devoted to finding strategies for the rational design and experimental stabilization of superhydrides with higher *T*_c_ and/or at reduced pressures, with an ultimate goal of near-ambient RTSC. In this regard, one of the promising approaches is to explore ternary or multinary superhydrides because they are expected to provide more abundant and diverse structure variants and chemical compositions. According to Flores-Livas *et al.*, assuming that ternaries can be formed with 60 elements of the periodic table, at a single pressure, not considering alloying, the number of possible, stable ternary hydride compounds is 1770 [2]. Again, theoretical predictions based on advanced computational algorithms play an important role in guiding this direction [4]. For instance, Sun *et al.* proposed an approach of designing new ternary high-*T*_c_ superhydrides via electron-doping into the binary superhydrides as exemplified in the clathrate-type Li_2_MgH_16_ with a remarkable high estimated *T*_c_ of ∼473 K at 250 GPa [5]. Another intriguing example is the ternary XYH_8_ structure containing H_24_ cages and H_8_ cubes; the combination of heavy X and light Y elements with disparate sizes enables this structure to exhibit more efficient stacking compared to previously explored binary clathrate structures. Remarkably, the *Fm-3m* phase of LaBeH_8_ has been predicted to be dynamically stable down to 20 GPa with a high *T*_c_ of ∼185 K [6].

Experimentally, encouraging progress has been achieved very recently. For example, by using (La, Ce) binary alloys as precursors, the *P6_3_/mmc*-(La, Ce)H_9_ was successfully synthesized at 113 GPa and its *T*_c_ is preserved to 176 K at ∼100 GPa, which is about 80 K higher than that of the binary La/Ce-H superhydrides at similar pressures [7]. A similar approach has also been applied to synthesize ternary (La, Y)H_10_ and (La, Al)H_10_ [8]. In these compounds, different metal cations are randomly distributed over the same crystallographic sites, and the disordered state with enhanced configuration entropy should have a significant effect on their stability and superconducting properties. Different from these pseudo-ternary hydrides, the successful synthesis of LaBeH_8_ at 110–130 GPa brought a ‘true’ stoichiometric ternary hydride, in which the BeH_8_ units and La are arranged orderly in a rocksalt-like structure. Under sub-megabar pressure of 80 GPa, this compound can preserve a moderately high *T*_c_ of 110 K [9].

The successful syntheses of these above-mentioned ternary superconducting hydrides rely on the selection and preparation of proper precursors. Currently, the disordered binary alloys (La, Ce/Y/Al/Be) obtained through high-temperature melting or magnetron sputtering have been used to synthesize the above ternary hydrides. The formation of disordered alloys requires a complete solubility of two elements at a specific ratio at high temperatures. In addition to the disordered alloys, the precursors can be in the forms of submicron mixtures and ordered intermetallic compounds made of two metal elements. Submicron mixtures can be obtained through ball-milling (mechanical alloying), cold/hot pressing, and magnetron sputtering, etc. These methods can prevent the premature reaction of the raw materials so as to avoid the formation of excessively stable phases, which could hinder further hydrogenation. On the other hand, the ordered intermetallic compounds with specific atomic ratio can be obtained via chemical reactions, giving rise to high homogeneity and precise chemical ratios at the atomic scale. These methods have their own merits and should be applicable for different elements. For example, the mechanical alloying at room temperature should be more feasible for the preparation of binary alloys containing highly reactive lithium, which could then be applied to synthesize the theoretically predicted Li_2_MgH_16_ [5].

To reduce the stabilization pressures of ternary hydrides to submegabar pressures is also helpful for settling down some critical issues about high-*T*_c_ superconductivity in hydrides [10]: (1) Due to the constraints of high-pressure techniques, most hydrides synthesized at megabar pressures contain multiphases, which poses challenges in identifying the superconducting phases and the accurate characterizations of their physical properties. As a result, decisive evidence of superconductivity, including zero resistance and Meissner effect, is lacking in most cases. (2) Since hydrogen has a small scattering cross section for X-rays, the crystal structure and chemical composition of superhydrides cannot be determined directly in experiments. Alternatively, indirect methods through a combination of theoretical calculations and volume-dependent measurements are adopted. (3) Characterizations of the microscopic properties in both superconducting and normal states under megabar pressure are very limited. To address these issues, further developments of *in-situ* high-pressure techniques for both samples’ syntheses and characterizations are highly desirable. For example, novel diffraction techniques using high-energy synchrotron radiation should be developed to analyze the structures of light-element compounds, and the micro-magnetic measurement techniques based on nitrogen-vacancy centers can be applied to detect the Meissner effect. We believe that these issues can be resolved soon accompanying further advancement of ternary superhydrides and high-pressure techniques.
